# Surface area and composition-engineered CoFe_2_O_4_/g-C_3_N_4_ hybrid electrodes for high-performance supercapacitors

**DOI:** 10.1080/14686996.2026.2700203

**Published:** 2026-07-16

**Authors:** Divakara S. G., Shwetha K. P., Mahesh B., Vishnumurthy K. A.

**Affiliations:** a Department of Chemistry, RV College of Engineering, Bengaluru, India; b Department of Physics, RV College of Engineering, Bengaluru, India; c Department of Chemistry, JSS Academy of Technical Education, Bengaluru, India

**Keywords:** Supercapacitor, hybrid electrode, CoFe_2_O_4_, g-C_3_N_4_, nano-composite, green synthesis

## Abstract

Efficient synthesis techniques coupled with enhanced specific capacitance are crucial for advancing the next generation of supercapacitor electrode materials. Here, we examine the electrochemical performance of hybrid electrodes for supercapacitor applications that involve graphitic carbon nitride (g-C_3_N_4_) reinforced with cobalt ferrite (CoFe_2_O_4_). CoFe_2_O_4_ nanoparticles were synthesized using an inexpensive and eco-friendly honey-mediated approach. These nanoparticles were combined with g-C_3_N_4_ with varying surface areas and evaluated as electrode materials for supercapacitors. The final composites were extensively characterized for morphology, structure, and electrochemical performance. According to the empirical results, a desirable CoFe_2_O_4_/g-C_3_N_4_ nanocomposite material with a proportion of 1:0.8 demonstrated a specific capacitance of 470 Fg^‒1^ at a scan rate of 2 mVs^‒1^ and 331 Fg^‒1^ at a current density of 0.5 Ag^−1^ within a potential range of 0 to 0.5 V in 2 M KOH. Heterostructures CoFe_2_O_4_/g-C_3_N_4_ (CoF/GCN_2_, ratio: 1:0.8) composites showed exceptional cyclic stability (80% of its capacitance after 10,000 cycles) at higher current densities (6 Ag^−1^), which was considerably greater than that of pristine g-C_3_N_4_, CoFe_2_O_4_, along with additional nanocomposite variations. It also achieved a power density of 2142.8 W kg^‒1^ and an energy density of 4.75 Wh kg^‒1^. It is believed that the higher electrochemical efficiency is largely attributed to the combined effect of porous g-C_3_N_4_ and CoFe_2_O_4_.

## IMPACT STATEMENT

The novelty of this work lies in the honey-mediated synthesis of CoFe_2_O_4_ nanoparticles followed by formation of a CoFe_2_O_4_/g-C_3_N_4_ heterostructure, exhibiting enhanced capacitance, rate capability, and cycling stability for supercapacitor applications.

## GRAPHICAL ABSTRACT

**Figure:**
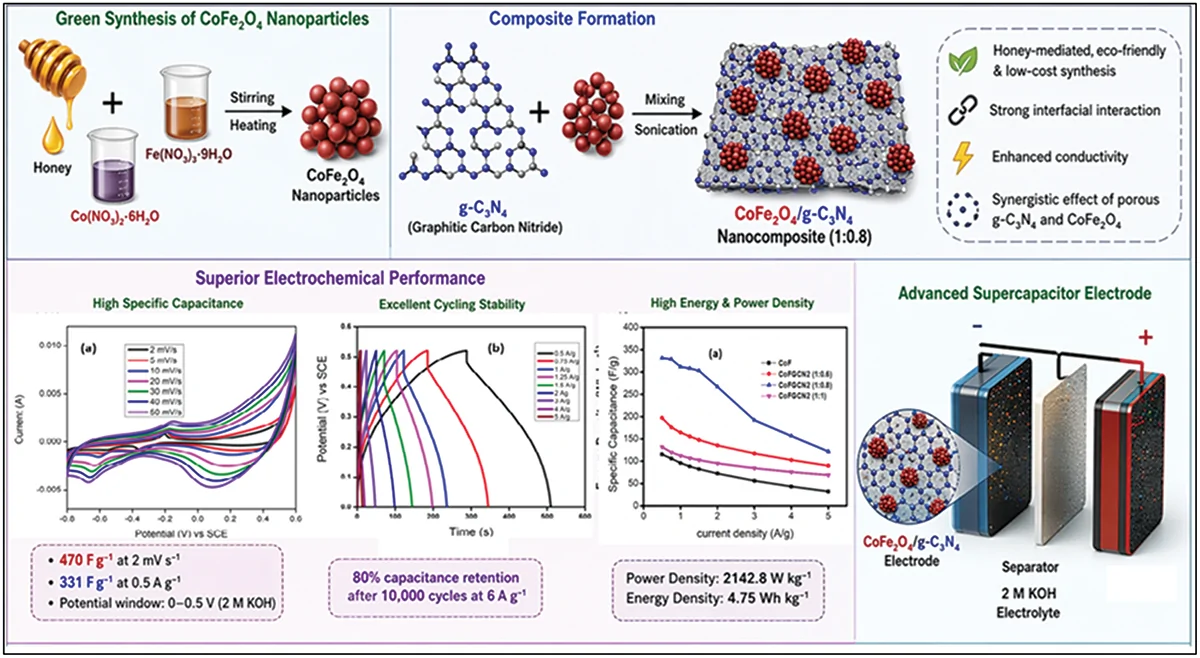

## 1. Introduction

High-performance energy storage systems are becoming increasingly important as clean energy technologies become a crucial component of sustainability [1]. Batteries have significantly advanced energy storage technologies; however, they suffer from certain limitations. Supercapacitors, in contrast, have garnered significant attention due to their superior electrochemical performance, including high cycle life, high power density, fast recharge and discharge rates, and reliable safety across a wide temperature range [2]. However, the energy density is lower than desired.

Recent years have seen numerous significant breakthroughs in next-generation supercapacitors [3]. These advancements include theoretical insights into their mechanisms, material synthesis, and the design of improved devices. A significant focus is on developing a fundamental understanding of the relationship between structural features and the electrochemical behavior of electrode materials. The physical and chemical characteristics of the electrode material significantly affect how effectively energy is converted and stored. Many variables determine supercapacitor performance, including the potential window, electrolyte, and electrode material. The selection of an ideal electrode for an efficient energy device depends on several factors, including its conductivity, chemical and thermal stability, and high surface area with porosity that provides an appropriate pore size [4]. Carbon materials, such as graphene, carbon fiber, carbon paste, carbon nanotubes, and activated carbon, hold significant promise owing to their high surface area, excellent conductivity, and configurable porous structures, which are crucial for enabling rapid ion motion and improving energy storage efficiency. For example, Zhang et al. demonstrated that CNTs can enhance electron mobility and electrolyte accessibility in supercapacitors, thereby increasing charge storage [5]. Despite these advantages, the energy density of carbon-based materials is often limited, hindering their use in high-energy devices [6].

Due to their high theoretical capacitance and redox activity, transition-metal-based composite materials, particularly oxides and chalcogenides, have significant potential as electrode materials. NiCo_2_O_4_ and MoS_2_, for instance, have been shown to notably advance energy storage performance in supercapacitors and batteries by enabling reversible redox reactions [7]. Nonetheless, these materials can exhibit cycling instability and mechanical degradation over extended use, especially in flexible or high-power applications [8]. By combining carbon materials with transition metal compounds (TMCs), researchers aim to synergize carbon’s conductivity and stability with TMCs’ high capacity [9]. However, the challenge remains to optimize these composites to balance performance, stability, and cost – an area where ongoing research continues to seek innovative solutions. Furthermore, these materials should be economical and environmentally friendly. Ongoing advancements in supercapacitor technology focus on improving the electrical performance of electrode materials, underscoring the importance of identifying or developing high-quality electrode materials for advanced supercapacitors.

Designing electrodes from transition metal oxides (TMOs), including binary, ternary, and complex metal oxides that exhibit pseudocapacitive behavior, has been the focus of extensive research for supercapacitor applications. These materials, along with metal ferrites, metal hydroxides, metal sulfides, metal oxynitrides, and conducting polymers, have attracted considerable attention due to their promising electrochemical properties [10,11]. Among these materials, particularly spinel ferrites (MFe_2_O_4_, where *M* = Mn, Co, Ni, or Cu) have emerged as a promising class of TMOs due to their unique combination of redox activity, structural flexibility, and cost-effectiveness. Their distinct crystal structure enables enhanced charge storage and transport, distinguishing them from other TMOs. This combination of properties including the ability to support different redox states, high power and energy densities, moderate electrical conductivity, and excellent stability-makes spinel ferrites strong candidates for next-generation supercapacitor electrodes [12]. The tetrahedral and octahedral sites in spinel ferrites, developed around a densely packed array of O^2−^ ions, are partially or fully occupied by M^2+^ and Fe^3+^ cations. These structures contain significant solid-state redox couples (Fe^3+^/Fe^2+^ and M^3+^/M^2+^), facilitating rapid charge transfer via hopping between cations of different valencies at low activation energy, thereby significantly enhancing the charge-storing capacity [13].

CoFe_2_O_4_, a widely studied spinel ferrite, has attracted significant interest ascribed to its high chemical and thermal stability, strong coercivity, high saturation magnetization, non-toxic and sustainable nature, and excellent electrical conductivity. In addition to the aforementioned properties, CoFe_2_O_4_ exhibits electrochemical performance in supercapacitors [14]. Its structural anisotropy, which enhances charge transport, and high theoretical specific capacitance, enabled by multiple redox-active sites, contribute to superior energy storage performance, making it a promising electrode material. However, further research and optimization are still needed to fully exploit its potential in practical energy storage devices.

One significant drawback of carbon-based electrode materials is their limited energy storage capacity, primarily because they rely on electrochemical double-layer capacitor (EDLC) mechanisms. The development of high-performance composite electrode materials, achieved by integrating carbon materials with conducting polymers or metal oxides, offers a solution to this limitation. The approach combines the high surface area of carbon for EDLC behavior with the faradaic charge storage mechanisms of metal oxides and polymers, resulting in significantly enhanced capacitance and energy density [15].

Graphitic carbon nitride (GCN) offers a unique alternative as a metal-free polymeric semiconductor with a layered structure resembling that of graphene [16]. The outstanding thermal and chemical stability of GCN, coupled with its easily available raw materials, and ease of synthesis make it highly desirable in various fields beyond energy applications [17,18]. GCN is an intriguing option for a range of applications, particularly supercapacitors, due to its high nitrogen content (C:N ratio of 3:4) and customizable characteristics [4,19]. These carbon-based compounds are essential for enhancing the electrode surface’s wettability and improving its electron-donating capabilities. However, the use of pure GCN in supercapacitor electrodes is constrained by its relatively limited surface area and poor electronic conductivity [20]. The combination of GCN with other materials, including metals, metal oxides, and polymers, has been the subject of substantial experimental and theoretical research during the last few decades. These efforts have significantly enhanced its capabilities, overcoming its inherent limitations. These challenges can potentially be addressed by preparing mesoporous g-C_3_N_4_ (mpg-C_3_N_4_), which offers a much larger surface area [21]. Previously, we demonstrated that heating melamine at 550°C with a small amount of ammonium carbonate or a small volume of Coca-Cola beverage as precursors increased the surface area. Consequently, the morphological changes and higher surface area obtained compared to pristine GCN resulted in superior electrochemical properties [22]. Moreover, integrating metal oxides, viz., nickel, cobalt, manganese, copper, and iron, into GCN to create nanocomposite electrodes markedly enhances the electrode’s surface area. This improvement occurs through the provision of additional active sites for reactions and the facilitation of ion diffusion throughout the electrode [20].

Despite the advantages of nanocomposite electrodes, the search for materials offering both high energy density and strong cyclic stability continues. This demand explores alternative materials and compositions for improved performance. Transition metal oxides, particularly those containing cobalt and iron, have attracted attention for their synergistic electronic and structural properties. Several researchers explored the fabrication of electrode materials combining spinel ferrites and GCN [23]. Ferrite-based composites, such as CoFe_2_O_4_/g-C_3_N_4_ (CoF/GCN), have attracted attention because of their distinct properties, including varied oxidation states, thermal and electrical properties and strong chemical stability. The structural stability and electrical conductivity of these composites, along with the synergistic effects within the hybrid structure, render them promising electrode materials for supercapacitor applications. They exhibit superior specific capacitance and cycle stability. Rani et al. created a CoFe_2_O_4_@g-C_3_N_4_ composite electrode using a glycol-mediated solvothermal process, achieving 157.5 Fg^−1^ at 2 Ag^−1^, surpassing bare CoFe_2_O_4_ (85 Fg^−1^ at 2 Ag^−1^) and retaining ~98% capacitance after 5000 cycles, showcasing excellent cyclic stability [24]. Hexaferrite-based materials, like BaCoFe_11_O_19_/g-C_3_N_4_ nanocomposites synthesized by Suganya et al., showed 94.3% capacitance retention after 10,000 cycles [25]. Yesmin et al. described a CoFe_2_O_4_/Cu/g-C_3_N_4_ hybrid exhibiting a specific capacitance of 1380 Fg^−1^ at 1 Ag^−1^, with 86.4% capacity retention after 10,000 cycles, and an energy density of 144.4 Whkg^−1^ at a power density of 7.992 kWkg^−1^ [26]. Investigating the synergy in materials like CoFe_2_O_4_ could improve energy storage technologies by improving performance and durability. Further studies on g-C_3_N_4_-CoFe_2_O_4_ compositions are required to explore their synergistic effects, particularly in ionic capacitance and retention, compared with pure GCN and CoFe_2_O_4_ electrodes. Green synthesis using biocompatible sources offers a sustainable method for the production of ferrite nanoparticles [27].

Honey, composed primarily of carbohydrates (80–85% glucose and fructose), serves as a natural fuel or template in the combustion synthesis of CoF nanoparticles [28]. Reducing properties of fructose, glucose, and vitamin C facilitate the formation of nanostructures by acting as capping agents, stabilizers, and chelators. These compounds bind metal ions, leading to smaller, more uniform nanoparticles and altering the spinel’s crystalline structure, thereby enhancing its physicochemical properties [29]. The honey-mediated green synthesis method offers a sustainable and efficient route to producing cobalt ferrite nanoparticles.

In the current work, CoF nanoparticles were synthesized using a honey-mediated auto-combustion method. The porous GCN was prepared by heating melamine at 550°C in the presence of ammonium carbonate and Coca-Cola beverages to enhance its surface area. Consequently, different morphologies of porous GCN were produced, which had a rapid effect on the material’s surface area and electrochemical features. The synthesis of hybrid materials by combining these GCN structures with varying mass ratios of CoF to study the synergistic effects on their electrochemical properties was investigated.

Nanocomposites with different mass ratios of GCN to CoF were successfully synthesized, and an optimum ratio of GCN/CoF composition for enhancing the supercapacitor performance was investigated for the first time. The incorporation of GCN onto CoF not only introduces nitrogen-containing carbon material but also facilitates the creation of superior structures, thereby enhancing electron transfer capabilities. The electrochemical behavior of CoF/GCN composites was investigated at various weight ratios. These results were compared with the performance of pristine GCN and CoF, and the findings are discussed. In comparison to samples of pure CoF and GCN, the CoF/GCN sample with the optimal CoF to GCN ratio (CoF/GCN2, 1:0.8) demonstrated enhanced electrochemical performance. A specific capacitance of 470 Fg^‒1^ was obtained at a current density of 0.5 Ag^‒1^. The CoF/GCN composite maintained 97.2% of its capacitance after 10,000 charge/discharge cycles at 6 Ag^‒1^, indicating excellent cycling stability.

## 2. Experimental section

### 2.1 Synthesis of bulk g-C_3_N_4_, porous g-C_3_N_4,_ mesoporous g-C_3_N_4_ and the CoFe_2_O_4_/g-C_3_N_4_ composites

#### 2.1.1 Synthesis of g-C_3_N_4_ (bulk GCN) and porous g-C_3_N_4_ (GCN1)

For GCN1, a 1:1 mixture of melamine and ammonium carbonate was thoroughly mixed and placed in a lidded silica crucible. The mixture was heated in a muffle furnace at 550°C for 4 hours, with a gradual temperature increase of 2–5°C per minute. To ensure complete conversion, the temperature was maintained at 550°C for an additional two hours. The resulting material was washed with ethanol, dried at 80°C, and characterized. Bulk GCN was prepared using the same procedure, excluding ammonium carbonate.

#### 2.1.2 Synthesis of mesoporous g-C_3_N_4_ (GCN2)

After mixing 10 g of melamine with 0.5 mL of Coca-Cola and 60 mL of deionized water, the mixture was sonicated for five minutes at room temperature. Then, the mixture was transferred to a 100 mL Teflon-lined autoclave and heated for 16 hours at 180°C. The autoclave was heated and then cooled to ambient temperature. After preparation, the sample was dried at 80°C. The material was then calcined for 4 hours at 550°C in air, with the temperature increasing by 2–5°C per minute. Subsequently, the substance was allowed to cool spontaneously to ambient temperature. The code for this sample was GCN2.

#### 2.1.3 Synthesis of CoFe_2_O_4_ (CoF) and CoFe_2_O_4_/g-C_3_N_4_ (CoF/GCN)

CoFe_2_O_4_ was synthesized by a simple auto-combustion process followed by a heat treatment. In 75 mL of distilled water, 4 mmol each of Fe(NO_3_)_3_•9 H_2_O and Co(NO_3_)_2_•3 H_2_O were dissolved, and the mixture was agitated for 30 minutes. The precursor metal salt solution was mixed with the 30 ml of honey solution, heated at 60°C for 60 min while stirring continuously, and then heated to boiling. At 90°C, the solution evaporated, causing the honey’s monosaccharides to break down. The solution changed throughout this period, transforming from sol to gel, leading to an explosion in the mixture and the formation of powder [30]. To prepare the hybrid nanomaterials, CoF and GCN2 powders were mixed in three weight ratios: 1:0.6, 1:0.8, and 1:1. The mixtures were then thoroughly ground to obtain a fine powder. The pulverized mixtures were then transferred to a furnace and calcined for 4 hours at 550°C as the last step. The resulting CoF/GCN hybrid samples were designated CoF/GCN2A, CoF/GCN2B, and CoF/GCN2C, corresponding to their initial CoF: GCN mixing ratios. The honey used in the study was procured directly from a beekeeper in the wild forests of Karnataka (Western Ghats), India, and was used without further purification.

### 2.2 Characterization

The materials were analyzed using monochromatic CuKα radiation with X-ray diffraction (XRD, Rigaku D/MAX-2000, Japan) and scanning electron microscopy (SEM, JEOL JEM-6380LV, APREO 2S, Japan). Using the Shimadzu IRAffinity-1SWL equipment (Japan), Fourier trans-form infrared spectra were captured. To measure nitrogen physisorption, a BET surface area analyzer (BELSORB MAX, Japan) was used. Electrochemical measurements were performed using a CHI 660E (USA) electrochemical workstation.

### 2.3 Electrochemical performance measurements

The comparison of the electrochemical performance of bulk GCN, porous GCN and the CoF/GCN composite was carried out using cyclic voltammetry (CV), chronopotentiometry (CP) and electrochemical impedance spectroscopy (EIS) on a CHI 660E electrochemical workstation with a three-electrode system in 2 M KOH electrolyte. The three-electrode system consists of a working electrode (Toray carbon paper coated with active material), a reference electrode (a Saturated Calomel electrode), and a counter electrode (a platinum wire). The working electrodes were fabricated by mixing 85 wt.% of active material with 10% of Ketjen black in a mortar along with a binder (5 wt.% of polyvinyldenedifluride dissolved in 1-methyl-2- pyrrolidinone- NMP). The prepared slurry was coated onto a pre-weighed current collector (Toray carbon sheet) with an area of 1 cm^2^. The electrodes were dried for 12 hours, and the remaining active mass was calculated. The electrochemical performance of the materials was studied in a three-electrode system and compared with respect to their Cs, ED, PD and cycle stability.

## 3. Results and discussion

### 3.1. Characterization of the GCN, GCN1, GCN2, and CoF/GCN2

**Figure 1.**
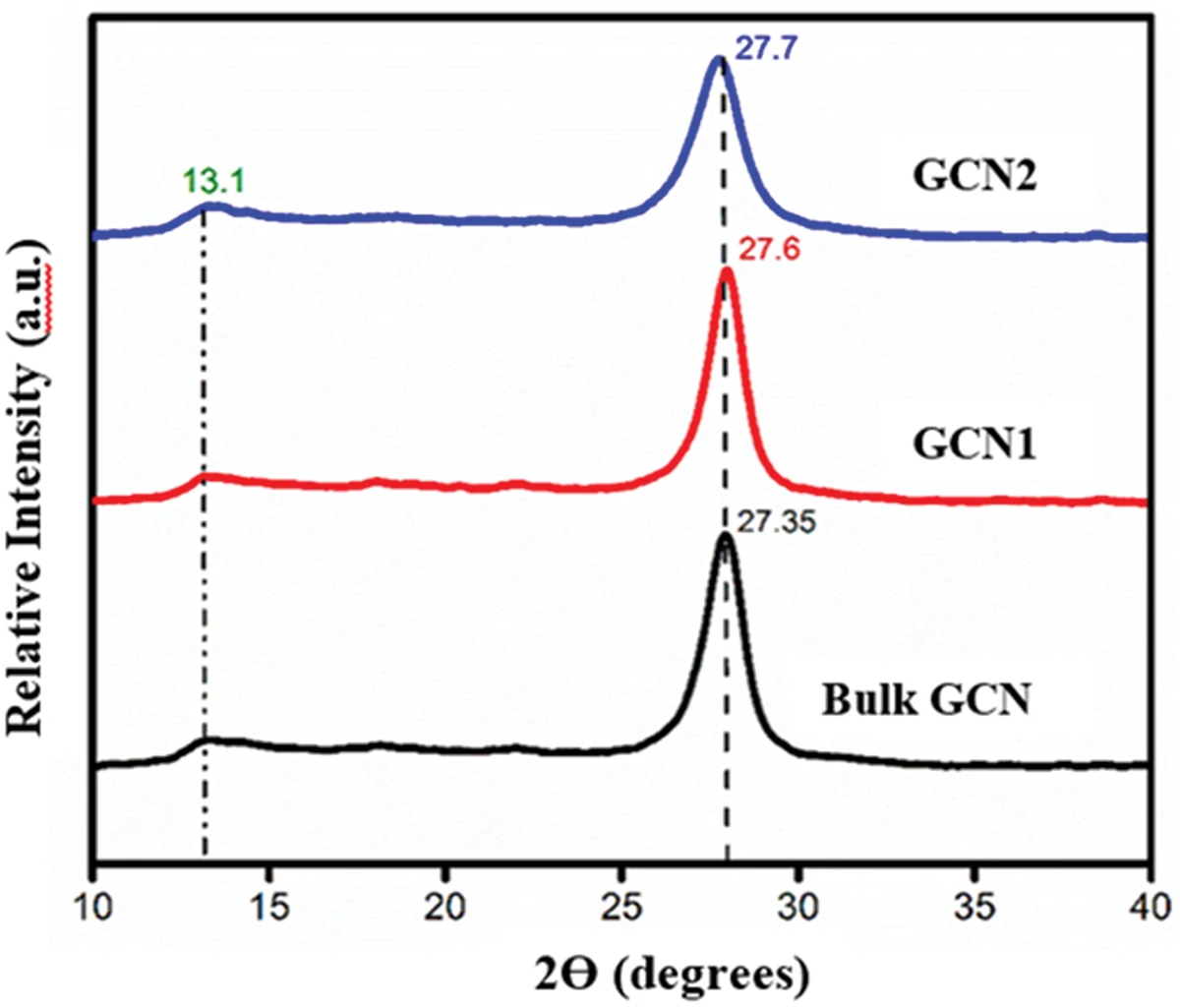
Indexed XRD patterns for bulk GCN, GCN1 and GCN2.

The structural properties of the three prepared samples, bulk GCN, GCN1, and GCN2 were analyzed using XRD in the 2θ range of 10–80°, while significant region of 10–40° is shown in Figure 1. The pattern shows two distinctive peaks at 13.1° and 27.3°, characteristic of GCN (JCPDS 87–1526). The minor peak at 13.1° corresponding to the hkl value of (100) represents the inter-planar spacing of 0.675 nm was observed. A more prominent peak at 27.3°, associated with the hkl value of (002), signifies a interlayer spacing of 0.325 nm, indicating a closer spacing between the sheets [31]. GCN1 and GCN2 each exhibited two stable peaks, representing that their crystal structures are fundamentally consistent with that of bulk GCN [22]. The structural packing pattern of the tris-s-triazine unit remained unchanged compared to bulk GCN, with carbon atoms arranged in a hexagonal honeycomb lattice in GCN. The spacing between these layers is indicated by the position of the (002) peak in the XRD pattern. Shifts and intensity changes in the C peak of XRD spectra provide valuable insights into the crystal structure and morphology of GCN materials prepared by different synthesis methods. Notably, the strong peak at 27.6° in GCN1 and 27.7° in GCN2 is slightly shifted compared to bulk GCN, corresponding to the (002) plane stacking of the conjugated aromatic system. The interlayer stacking distance in GCN can be affected by various structural characteristics, including the presence of impurities or structural defects, the degree of crystallinity, and the incorporation of functional groups on the surface of the GCN layers. These factors can cause the layers to be stacked either closer together or further apart, leading to a shift in the position of the (002) peak. For GCN1 nanosheets prepared through an exfoliation process using ammonium carbonate, the (002) peak shifts from 27.3° to 27.6°, indicating a reduction in the interlayer spacing. This decrease is likely due to the formation of thinner nanosheets during the exfoliation process, which brings the layers closer together. Ultrathin nanosheets often exhibit a shift of the (002) peak to higher angles, along with an increase in the interlayer gap. This is because the exfoliation process can disrupt the original stacking of the bulk GCN layers, leading to a more disordered arrangement. Additionally, functional groups on the surface of the GCN layers can introduce steric repulsion, forcing the layers to stack further apart. Consequently, as the interlayer space enhances, the (002) peak shifts to higher angles. In the case of GCN2 nanosheets prepared through an exfoliation process using Coca-Cola treatment, the (002) peak shifts to a higher angle (around 27.7°). This shift suggests a decrease in the interlayer distance in the nanosheets compared to the other two samples [32]. S. Sun et al. emphasized the effect of different sugars on the exfoliation and structural transformation of g-C_3_N_4_, resulting in the development of an exceptionally porous material [33]. Furthermore, the lower diffraction intensity of the GCN2 peak compared to pristine GCN suggests a reduction in overall crystallinity and a less ordered arrangement of atoms within the nanosheets. This loss of long-range order likely arises from the disruption of in-plane hydrogen bonds and the twisting of polymeric melon units during the high-temperature exfoliation [34].

**Figure 2.**
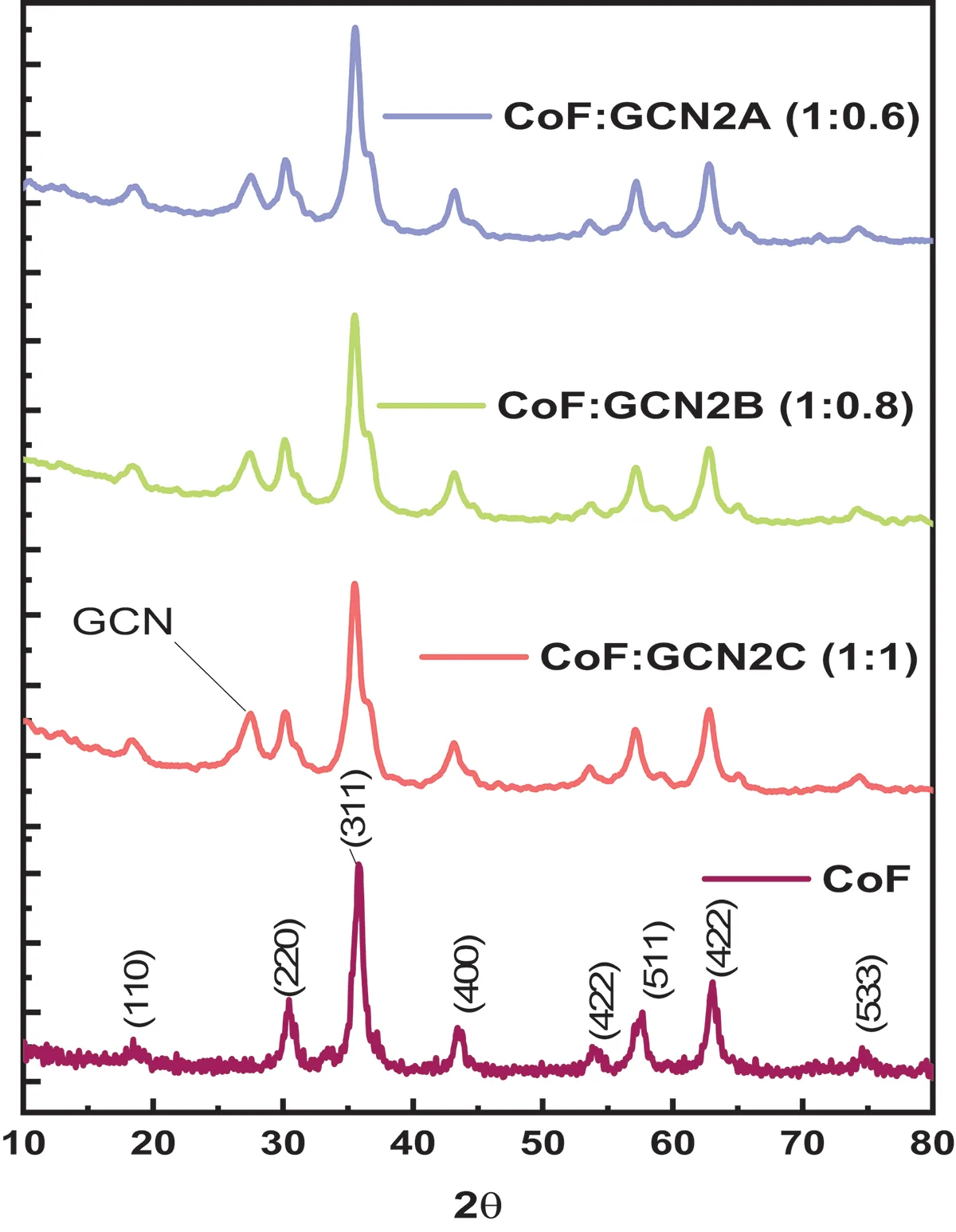
XRD patterns for the CoF and CoF/GCN2 composites with varying ratios.

XRD analysis was used to determine the phase composition and structural properties of CoF/GCN nanocomposites featuring various mass ratios, as illustrated in Figure 2. The figure depicts the XRD patterns obtained for both cobalt ferrite nanoparticles and CoF/GCN2 composites, with differing proportions of CoF to GCN2. GCN2 was chosen as graphitic carbon nitride due to its higher surface area, characterized by mesoporous architecture. The indexed XRD pattern of the prepared CoF nanoparticles and all peaks evidenced at 2θ angles of 30.3°, 35.5°, 37.1°, 43.2°, 53.6°, 57.2°, 62.8°, and 72° can be attributed to the (220), (311), (222), (400), (422), (511), (422), and (533) planes of cubic CoFe_2_O_4_ (JCPDS no. 22–1086) respectively. The broadening of the diffraction peaks confirms the formation of nanostructured CoF. It was observed that the average crystallite size of CoF was 6 nm. The pattern of the CoF/GCN2 prepared composite sample reveals a satisfactory polycrystalline CoF and is completely compatible with the characteristic peaks of the cubic spinel structure [35]. The presence of characteristic peaks from both GCN and CoF asserts the concurrence of these phases without any additional impurity phases. Sharp diffraction peaks at 27.6° were marked in the XRD patterns of CoF/GCN2, ascertaining that the structure of GCN remained unchanged. The matching typical peaks were noticed in the XRD patterns of prepared CoF/GCN2B and CoF/GCN2C composite samples. As noticed in Figure 2, there are no other peaks related to cobalt oxide, iron oxide, or different phases, reflecting that these composites consist of GCN and CoF phases only. As the GCN content rises in the composites, the intensity of the (002) diffraction peak gradually increases from CoF/GCN2A to CoF/GCN2C, consistent with the incremental increase in GCN loading across the three weight ratios. The relative difference in GCN mass fraction between the three composites is modest (0.6 to 1.0 parts per unit CoF), so the change in absolute GCN peak intensity is incremental rather than pronounced. The dominant CoF spinel peaks retain high intensities throughout. Simultaneously, a slight increase in the (002) peak’s lattice spacing from 27.3° to 27.5° is observed. These findings suggest a close interaction between CoF nanoparticles and GCN nanosheets, altering the growth conditions of GCN and leading to the formation of CoF/GCN nanocomposites [36]. The average crystallite size of CoF/GCN2, determined with the Debye-Scherer formula, was calculated to be 8 nm. Calculated value of lattice parameter of CoF/GCN2 sample is 8.376 Å, which is in close agreement with standard data (8⋅388 Å).

**Figure 3.**
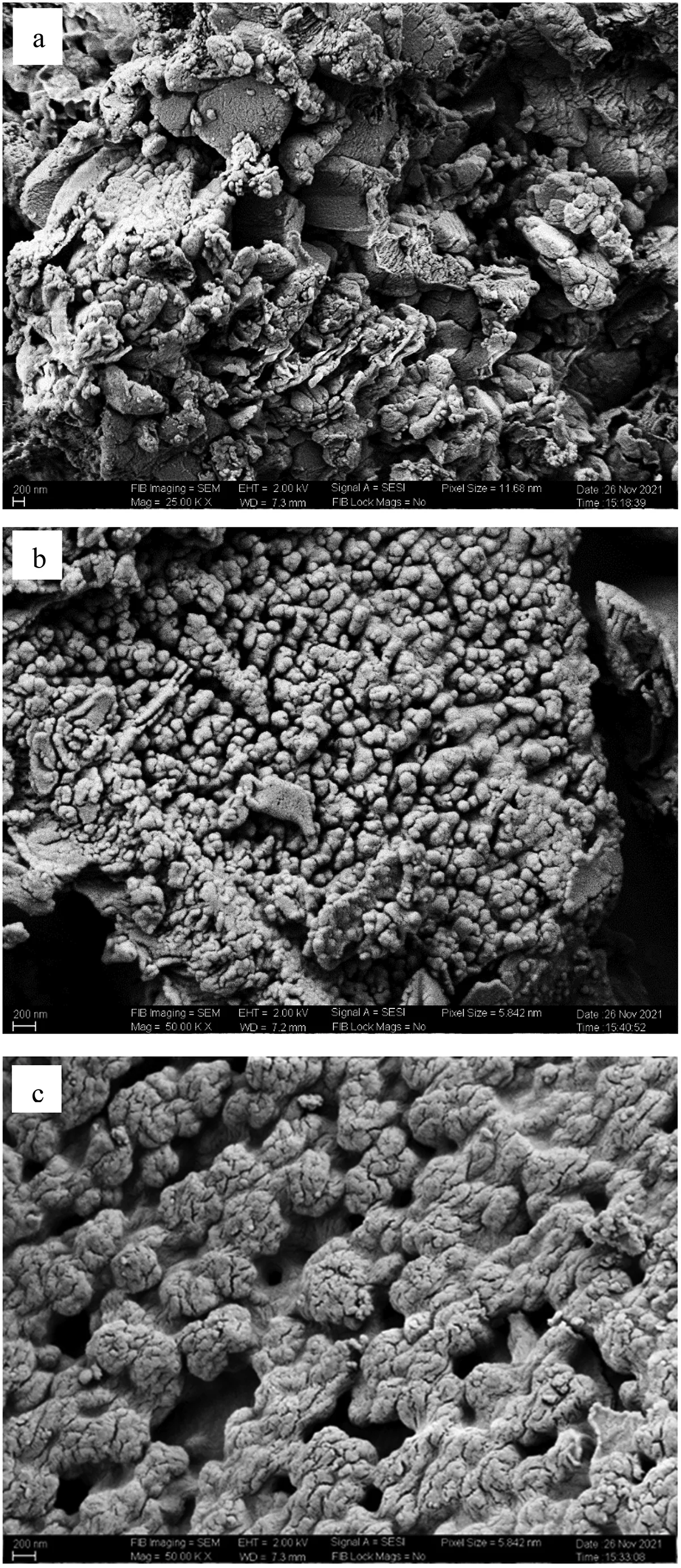
FESEM image of a) bulk GCN, b) GCN1, and c) GCN2.

The morphologies of the prepared bulk GCN, GCN1, and GCN2 samples were examined using FESEM, as shown in Figure 3(a–c) respectively. The bulk GCN was found to have a layered sheet-like structure. In GCN1, the stacked structure of bulk GCN is largely replaced by a more porous closed structure, with notable mesopores, as illustrated in Figure 3(b). The surface of GCN2, a lamellar sheet with numerous holes smaller than a nanometer in diameter and an unusual curled-closed structure with multiple pores, is portrayed in Figure 3c. Melamine was hydrothermally treated with carbon beverages, resulting in a remodeled microstructure and an extremely porous framework in the GCN2 material. Sugar plays a vital role in influencing the structural properties of GCN [37]. The exfoliation process, facilitated by the sugar content in Coca-Cola, results in a highly porous GCN2 structure with a significantly increased surface area, as evidenced by SEM images.

The exfoliation of GCN2 in the presence of Coca-Cola is primarily attributed to its sugar content, mainly sucrose and its hydrolysis product glucose. These small hydroxyl-rich organic molecules are capable of penetrating the interlayer space of GCN2 through hydrogen-bonding interactions with the surface –NH and –NH_2_ groups. This intercalation weakens the van der Waals forces that hold the stacked GCN layers together during the subsequent thermal treatment [32]. During the calcination step, the intercalated sugar molecules undergo pyrolytic decomposition, generating a significant volume of gaseous byproducts such as CO_2_ and H_2_O. This in-situ gas evolution acts as chemical blowing agent, driving mechanical separation of the GCN layers [37]. Additionally, the acidic nature of Coca-Cola promotes protonation of surface amine groups, thereby enhancing electrostatic repulsion between layers and further facilitating delamination. The synergistic combination of these mechanisms, results in highly porous architecture and increased specific surface area of GCN2 observed in the SEM analysis.

**Figure 4.**
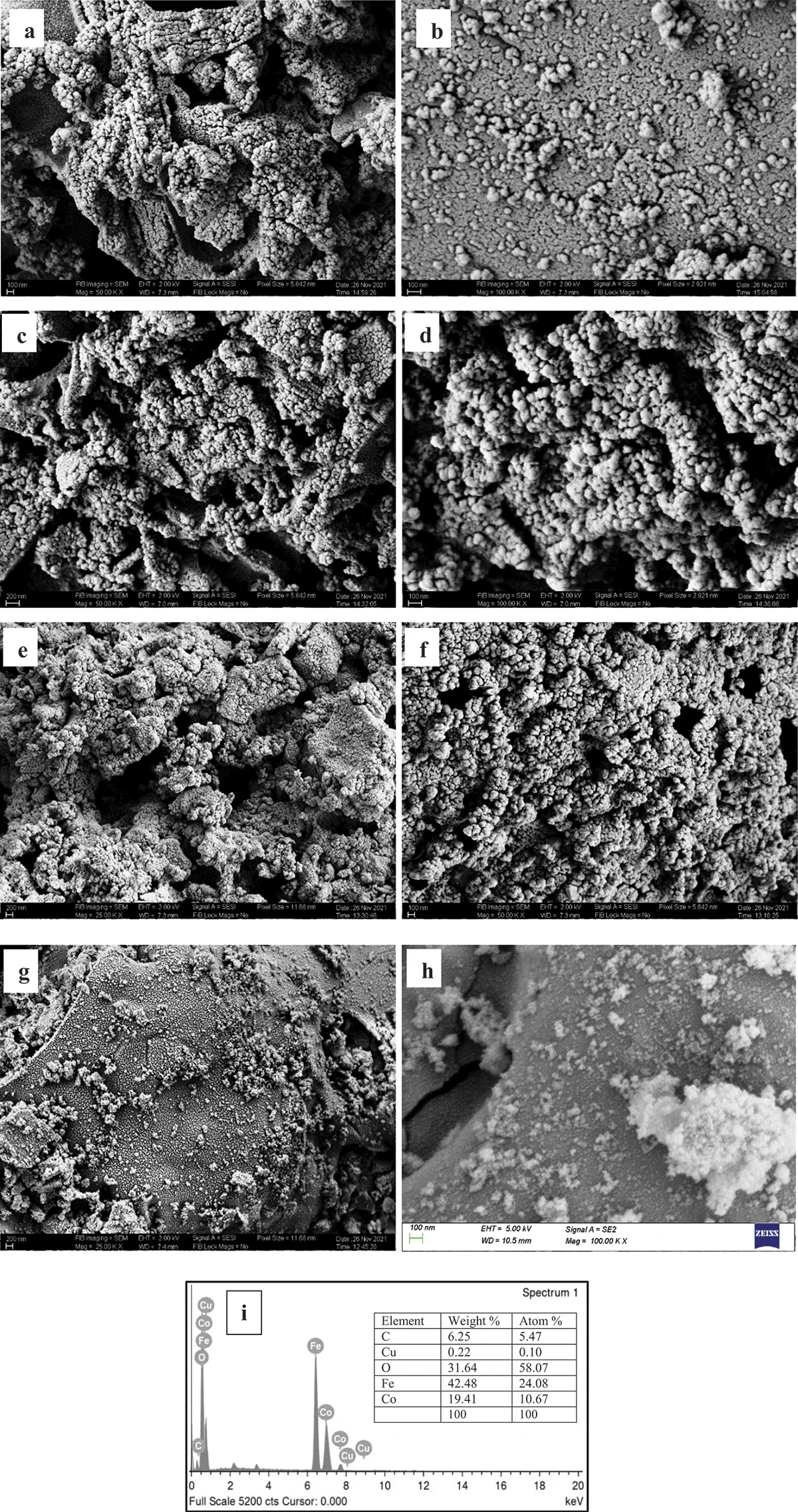
SEM images of CoF/GCN2 composites prepared at different ratios of mixing (a, b) 1:0.6 (CoF/GCN2A), (c, d) 1:0.8 (CoF/GCN2B), (e, f) 1:1 (CoF/GCN2C), (g, h) SEM images of CoF calcined at 550°C, i) energy dispersive X-ray spectroscopy (EDS) of CoF.

**Figure 5.**
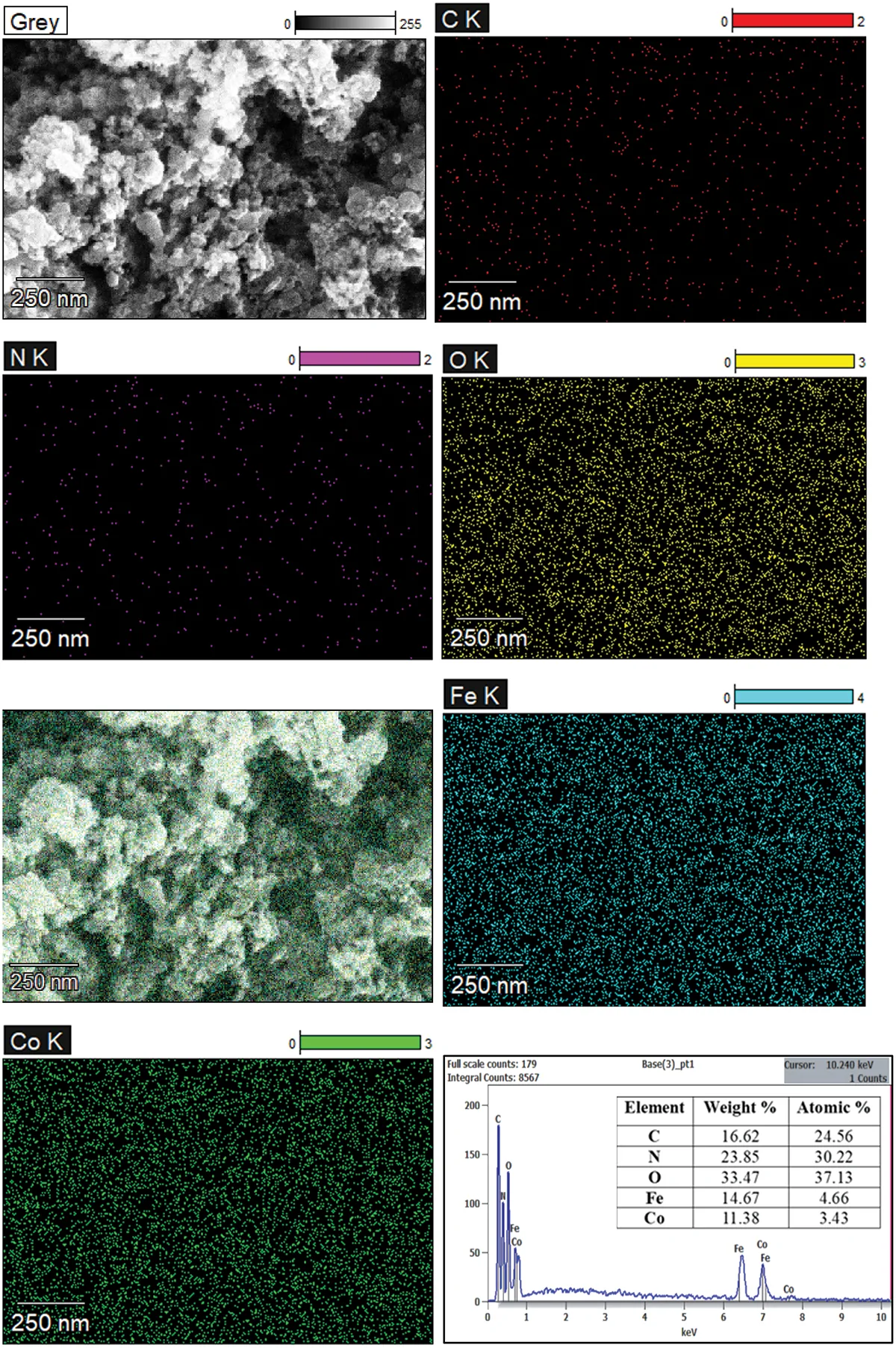
SEM micrograph of the area selected for EDS analysis, corresponding elemental mapping images, and EDS spectrum of the representative CoF/GCN2B hybrid sample.

The microstructures and surface morphology of composites prepared by mixing and calcining GCN2 with CoF at different ratios were examined using FESEM. In Figure 4(a-f), SEM images of CoF/GCN2A, CoF/GCN2B, and CoF/GCN2C reveal that GCN2 nanosheets were effectively decorated with CoF nanoparticles. The CoF nanoparticles attained a spherical shape with slight agglomeration and were well deposited on GCN. The increased surface area and the magnetic forces between the nanoparticles may contribute to the formation of small agglomerations. The SEM image of CoF sample in Figure 4(g,h) shows nearly spherical nanoparticles with an average diameter of about 12 nm. The EDS spectra for CoF in Figure 4(i) confirmed the presence of Co, Fe, and O by the peaks of elements at their corresponding keV values. Compositional analysis of GCN/CoF was also carried out using EDS. The EDS spectra of the CoF/GCN2B sample are shown in Figure 5. During the measurements, different regions were examined to estimate the elemental composition of oxygen, nitrogen, carbon, cobalt, and iron, and a summary of the elemental distribution is provided. The characteristic peaks observed in the EDS spectra confirm the presence of O, C, N, Co, and Fe in the hybrid sample.

The presence of Co, Fe, and O clearly confirms the formation of the ferrite phase, while the presence of C and N confirms the GCN in the hybrid sample. No additional impurity peaks were detected, indicating the high purity of the synthesized sample. The inset table presents the calculated atomic percentages of Co, Fe, C, N, and O. Furthermore, elemental mapping analysis performed on a randomly selected region reveals the uniform spatial distribution of Co, Fe, N, C, and O, confirming the homogeneous composition of the synthesized hybrid sample (Figure 5). The SEM images shown in Figure 5 correspond to the same field of view used for EDS mapping and the associated elemental distribution maps, enabling a direct comparison between the surface morphology and elemental distribution of the sample.

The honey-mediated synthesis approach employed in this study was adopted from previously reported literature [38]. Honey is primarily composed of simple sugars such as fructose and glucose, which together constitute nearly 60–75% of the total sugar content, while disaccharides and oligosaccharides contribute approximately 5–15%. In addition to fructose and glucose, several other saccharides, including sucrose, maltose, turanose, palatinose, and isomaltose, have also been identified in honey. Apart from sugars, honey contains a wide range of bioactive constituents such as proteins, amino acids, phenolic compounds, flavonoids, vitamins, and enzymes. Owing to the presence of these biomolecules, honey can simultaneously act as a reducing, stabilizing, capping, chelating, and fuel agent during nanoparticle synthesis. The hydroxyl-rich structures of glucose and fructose facilitate electron donation, thereby promoting the reduction of metal ions and suppressing nanoparticle agglomeration.

During the honey-mediated synthesis of ferrite nanoparticles, the metal salts dissociate to release positively charged metal ions, which subsequently interact with the hydroxyl groups of fructose and glucose, initiating nucleation and nanoparticle growth [30]. Furthermore, the viscous nature of honey provides a protective medium that supports controlled particle formation. During calcination, the organic constituents present in honey contribute to the development of porous morphology and improved crystallinity of the CoF nanoparticles. Variations in the sugar and phenolic composition of honey may further influence the morphology and structural properties of the synthesized nanoparticles.

FTIR spectroscopy was employed to analyze the substituent groups of the molecules prepared in the GCN, GCN1, and GCN2 samples in Figure S1 (Supplementary Information). As shown in Figure S1, the FTIR spectrum shows all the characteristic bands of pure GCN phase. A broad spectral band observed between 3100 and 3300 cm^−1^ is attributed to the stretching vibrations of unreacted primary (–NH–) and secondary (–NH_2_) amines located at the edges of the graphitic carbon nitride heterocycles. Multiple absorption peaks within the 1200–1720 cm^−1^ range correspond to the stretching vibrations of C–N and C=N bonds within the aromatic CN repeating unit [39]. The absorption peaks at 880 and 806 cm^−1^ confirm the characteristic out-of-plane vibrational modes associated with the triazine/s-triazine aromatic rings.

**Figure 6.**
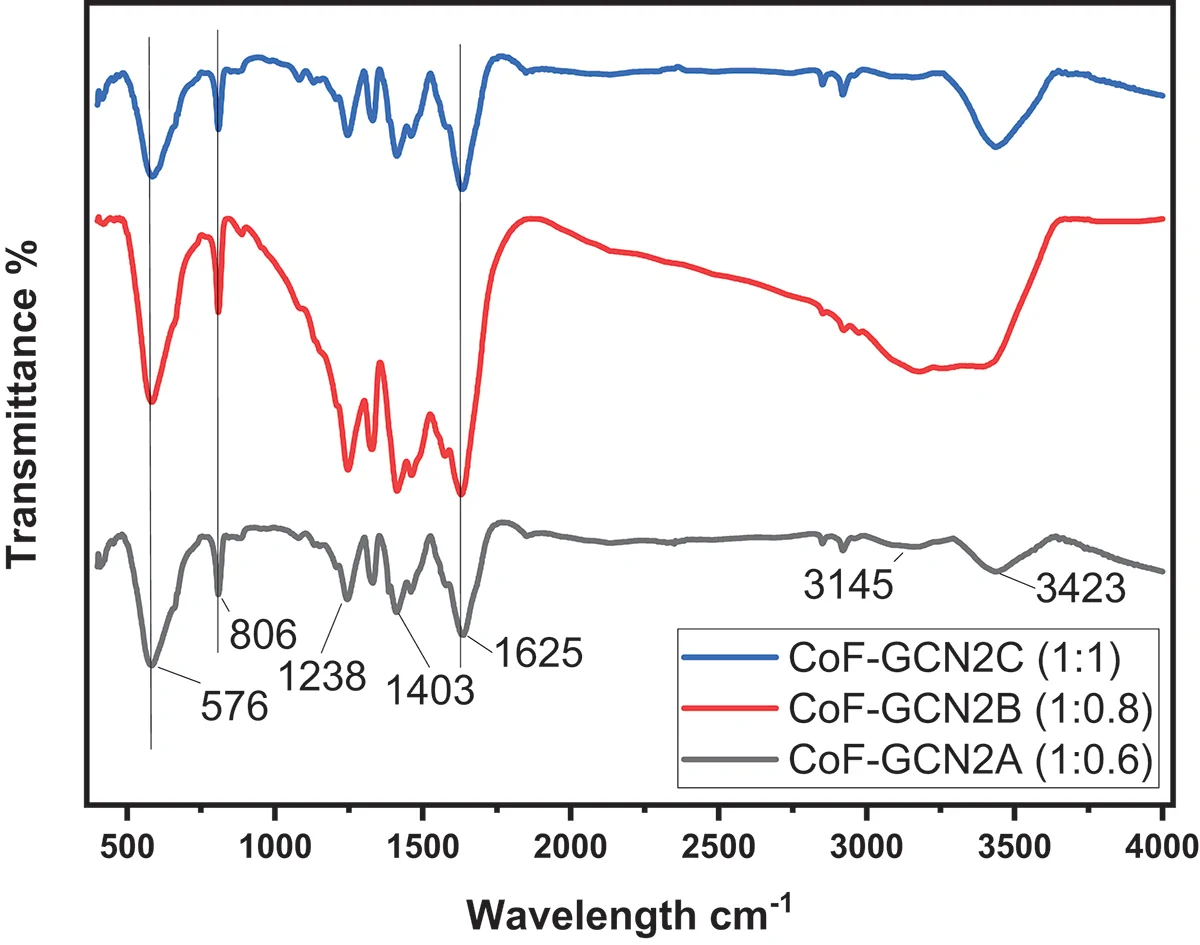
FTIR spectra of CoF/GCN2 composites prepared at different ratios.

FTIR spectroscopic analysis was carried out on the prepared CoF/GCN2 composite samples. Figure 6 presents the IR spectra of the CoF/GCN2 composite sample. The distinctive breathing vibration of s-triazine units is responsible for the prominent band at exactly 800 cm^−1^ [40]. The secondary and primary amines at the defect sites of the aromatic rings are associated with the broad spectrum observed around 3000–3500 cm^−1^, where the absorption peak intensities increase. Additionally, multiple bands within the 1100–1700 cm^−1^ range can be linked to the characteristic stretching modes of C–N heterocycles present in the GCN component of the composite. The distinctive pattern near 576 cm^−1^ in Figure 6 is attributed to the stretching vibration of the Fe–O bond in spinel-type CoF nanoparticles. FTIR spectra of the CoF/GCN2A, CoF/GCN2B, and CoF/GCN2C nanocomposites exhibit characteristic bands attributed to GCN at 1100–1650 and 810 cm^−1^, in addition to a strong band associated with CoF at approximately 580 cm^−1^. This spectral evidence confirms the successful integration of GCN and CoF components within the synthesized nanocomposites.

Surface area and pore size are widely recognized as key determinants of electrode material properties. Surface area, pore volume, and pore size distribution were determined for the GCN samples prepared using different precursors, and results are presented in Figure S2 (Supplementary Information). GCN2 shows a well-ordered mesoporous material with pores measuring 4.5 nm in diameter. Compared to bulk GCN and GCN1, GCN2 exhibited a superior surface area of 68.66 m^2^ g^−1^ versus 38.77 m^2^ g^−1^ and 53.22 m^2^ g^−1^, respectively. Additionally, GCN2 demonstrated a larger pore volume of 0.209 cm^3^g^−1^, surpassing the values of GCN1 (0.156 cm^3^g^−1^) and bulk GCN (0.136 cm^3^g^−1^). GCN2’s broad pore size distribution, high surface area, and high pore volume collectively likely to contribute to its exceptional performance as an electrode material compared to other GCN samples.

**Figure 7.**
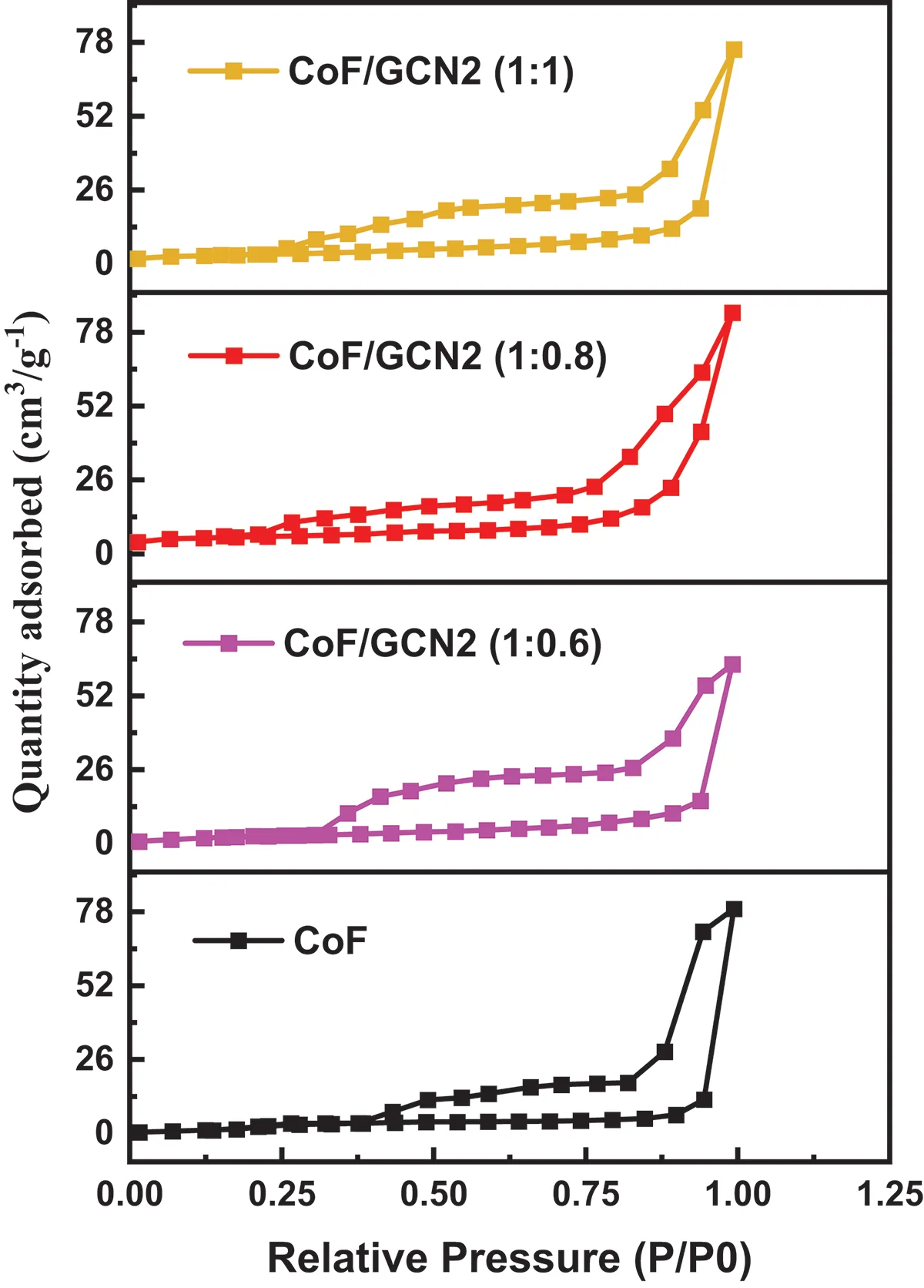
Nitrogen adsorption-desorption isotherms of investigated hybrid samples.

The surface properties of the prepared CoF/GCN2 nanocomposite samples were subjected to determine the surface area, pore volume, and corresponding pore-size distribution curve using the BET technique. The mesoporous structure of electrode material enhances supercapacitor performance by providing a considerable electrode-electrolyte interface for efficient charge storage and facilitating rapid ion diffusion through shortened pathways. Higher surface area and pore volume could be contributing factors to the improved electrochemical response. As shown in Figure 7, for all the composite samples, the N_2_ adsorption – desorption isotherms display a typical type-IV curves with H4 type hysteresis loop according to IUPAC, which is characteristic of the porous material in which the adsorption proceeds via multilayer adsorption followed by capillary condensation. Table 1 outlines the texture properties of the examined samples obtained from N_2_ adsorption/desorption isotherms, including BET surface area, average pore diameter, pore volume, and pore size distribution computed using the BJH method. It can be seen that, CoF/GCN2B has a smaller pore diameter (1.974 nm), higher pore volume (0.139 cm^3^g^−1^), and larger surface area (54.211 m^2^ g^−1^), compared to CoF/GCN2A CoF/GCN2C. This suggests that CoF/GCN2B’s structure likely consists of narrowly distributed pores, which enhance the surface area while maintaining a significant cumulative pore volume. Due to these characteristics, CoF/GCN2B is an excellent choice for an electrode in supercapacitors, where rapid charge-discharge cycles and efficient energy storage depend on a high surface area and optimal pore structure. The outcome advocates that the ratio of CoF to GCN maintains a significant influence on surface attributes. When CoF NPs were loaded, the BET surface area and pore volume of GCN2 decreased to 54.2 m^2^/g and 0.13 cm^3^/g, individually, suggesting that the CoF NPs formed a CoF/GCN2B mesoporous nanocomposite.

**Table 1.** The textural parameters of GCN, CoF and CoF/GCN2 composite of different ratios.

Sample	Pore volume (cm^3^g^‒1^)	Pore size (nm)	BET Surface area (m^2^g^‒1^)
Bulk GCN	0.136	3.109	38.77
GCN1	0.156	3.199	53.22
GCN2	**0.209**	**4.5**	**68.66**
CoF	0.146	1.932	72.407
CoF/GCN2A (1:0.6)	0.075	4.578	15.679
CoF/GCN2B (1:0.8)	**0.139**	**1.974**	**54.211**
CoF/GCN2C (1:1)	0.097	4.471	15.444

### 3.2 Electrochemical studies

**Figure 8.**
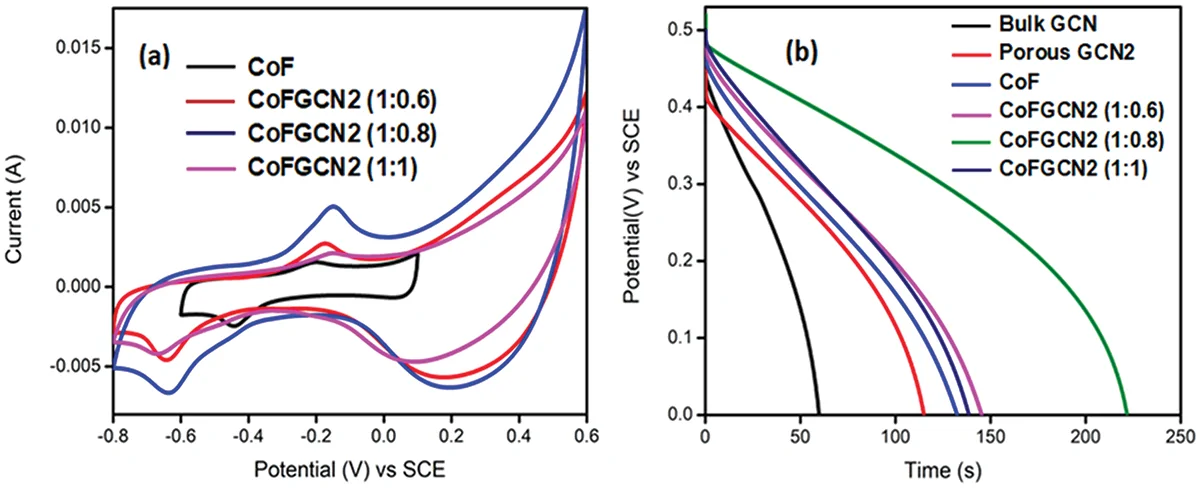
(a) CV curves of the CoF and the CoF/GCN2 hybrid electrodes at a scan rate of 2 mV s^−1^ in 2 M KOH, and (b) GCD curves at current density 0.5 Ag^−1^.

**Figure 9.**
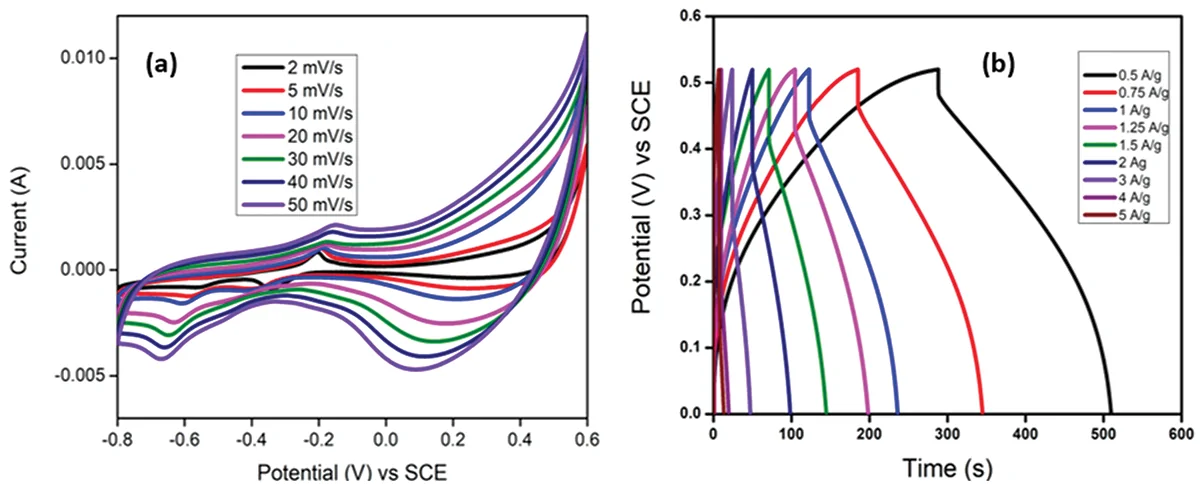
(a) CV curves of COF/GCN2B at various scan rates (2 to 50 mV s^−1^) in 2 M KOH; (b) GCD curves of COF/GCN2B at various current densities (0.5 to 5 a g^−1^).

Evaluating supercapacitor performance involves considering its specific capacitance (Fg^−1^, denoted as C_sp_), equivalent series resistance (ESR), and life cycle performance. Cyclic voltammetry (CV) and galvanostatic charge-discharge (GCD) measurements were employed to examine the electrochemical performance and charge storage mechanism of GCN, CoF, and CoF/GCN2 composite nanoparticles. Cyclic voltammetry tests were conducted to establish an appropriate operational voltage range for the electrodes and validate any faradaic processes during charging. Figure 8(a) shows the CV profiles of CoF and CoF/GCN2 composites measured in a 2 M KOH solution at a scan rate of 2 mV/s over a potential range of −0.8 to 0.6 V. With the different ratios of GCN, well-distinguished redox peaks in CoF/GCN2 nanocomposites shows excellent pseudo capacitance behavior of the composite. As shown in Figure 8(a), CoF/GCN2 hybrid electrodes exhibit a higher redox peak current and larger integrated area compared to the CoF supercapacitor electrode. The supercapacitor capabilities of CoF/GCN have shown that hybrid nanostructures demonstrate superior performance compared to their counterparts and their respective base materials. This indicates that CoF/GCN supercapacitor electrodes offer a broader operational voltage range, making them advantageous for high-voltage energy storage applications. The highest response and therefore the best capacitive performance were observed for the CoF/GCN2B (1:0.8) sample at the given scan rate. The potential use of CoF/GCN2 nanocomposites as electrode materials for supercapacitors was evaluated using GCD measurements, as shown in Figure 8(b). The CV of CoF/GCN2B nanoparticles is shown in Figure 8(a) for scan rates ranging from 2 to 100 mV/s in the potential range of −0.8 to 0.6 V. The quasi-rectangular shape of the CV curves indicates the composites’ capacitive behavior. The increased capacitance can be attributed to the substantial surface area and well-defined hetero-interface between the CoF and GCN2. This observation is consistent with the BJH measurements, which revealed a greater number of accessible pores in CoF/GCN2B, facilitating ion adsorption. The CV curves retain the shape up to scan rates of 2 to 50 mV/s, as shown in Figure 9(a), indicating good rate capability and a fast response in the aqueous electrolyte. These results highlight an increase in electrochemical activity and an enhanced reaction rate for the composite material. The observed increase in CV curve area with increasing scan rates, suggests a pseudocapacitive behavior. This pseudocapacitive mechanism is likely due to electrochemical adsorption and desorption processes at the interface between the electrode and the electrolyte. The specific capacitance by CV and GCD curves were calculated using the equations [41],

(1) $$C_{\mathrm{sp}}=\int \frac{I.\vartheta .\mathrm{\Delta V}}{m}\left(\mathrm{By}\;\mathrm{CV}\;\mathrm{graph}\right)$$

(2) $$C_{\mathrm{sp}}=\frac{I\;x\;\Delta \;t}{\Delta V.m}\left(\mathrm{By}\gcd\mathrm{curves}\right)$$

Where C_sp_ (F/g) represents the specific capacitance, I (A) denotes the charge-discharge current, m (g) signifies the mass of the active material, ΔV (V) indicates the potential range, ϑ (mVs^−1^) is the scan rate, and Δt (s) represents the discharging time.

The GCD curves of the CoF/GCN2 nanoparticle electrode at different current densities, ranging from 0.5 to 5 A/g, are shown in Figure 9(b). These curves demonstrate high charge-discharge coulombic efficiency and minimal polarization, as they are nearly symmetrical at all current densities [42]. Specific capacitances derived from GCD curves at 0.5Ag^−1^ are presented in Table 2 for different material compositions.

**Table 2.** Electrochemical properties of bulk GCN, porous GCNs, CoF, and CoF/GCN2 composites.

Electrode material	Specific Capacitance (CV) in Fg^−1^ at scan rate 2 mVs^−1^	Specific Capacitance (GCD) in Fg^−1^at 0.5 Ag^−1^	Energy density (WhKg^−1^)	Power Density (WKg^−1^)	Columbic efficiency after 5000 cycles %	Capacitance retention %
Bulk GCN	205	64.78	1.14	250.0	86	75
Porous GCN1	229	218.9	3.96	125.0	96.4	90
CoF	136	115	3.47	1562.5	95.5	83
CoF/GCN2A	301	197	3.3	1857.14	96.5	92
CoF/GCN2B	**470**	**331**	**4.75**	**2142.86**	**97**	**95**
CoF/GCN2C	390	132	2.875	1500	89.8	92

The CV curve of bare GCN is not shown in Figure 8(a) for clarity, as it exhibits a significantly lower current response and nearly rectangular EDLC behavior without discernible redox peaks. The pseudocapacitive properties of the CoF and CoF/GCN composites would be obscured if presented in the same graph. Table 2 delineates GCN’s electrochemical performance individually and analyzes it for comparative purposes.

The comparison clearly shows that CoF/GCN2B has the greatest C_sp_ value. The specific capacitance of CoF/GCN2B (470 Fg^−1^) was higher than that of CoF/GCN2A (301 Fg^−1^) and three times higher than pure CoF (136 Fg^−1^). This substantial improvement highlights the importance of mesoporous surface area in the CoF/GCN2B (1:0.8) hybrid.

**Figure 10.**
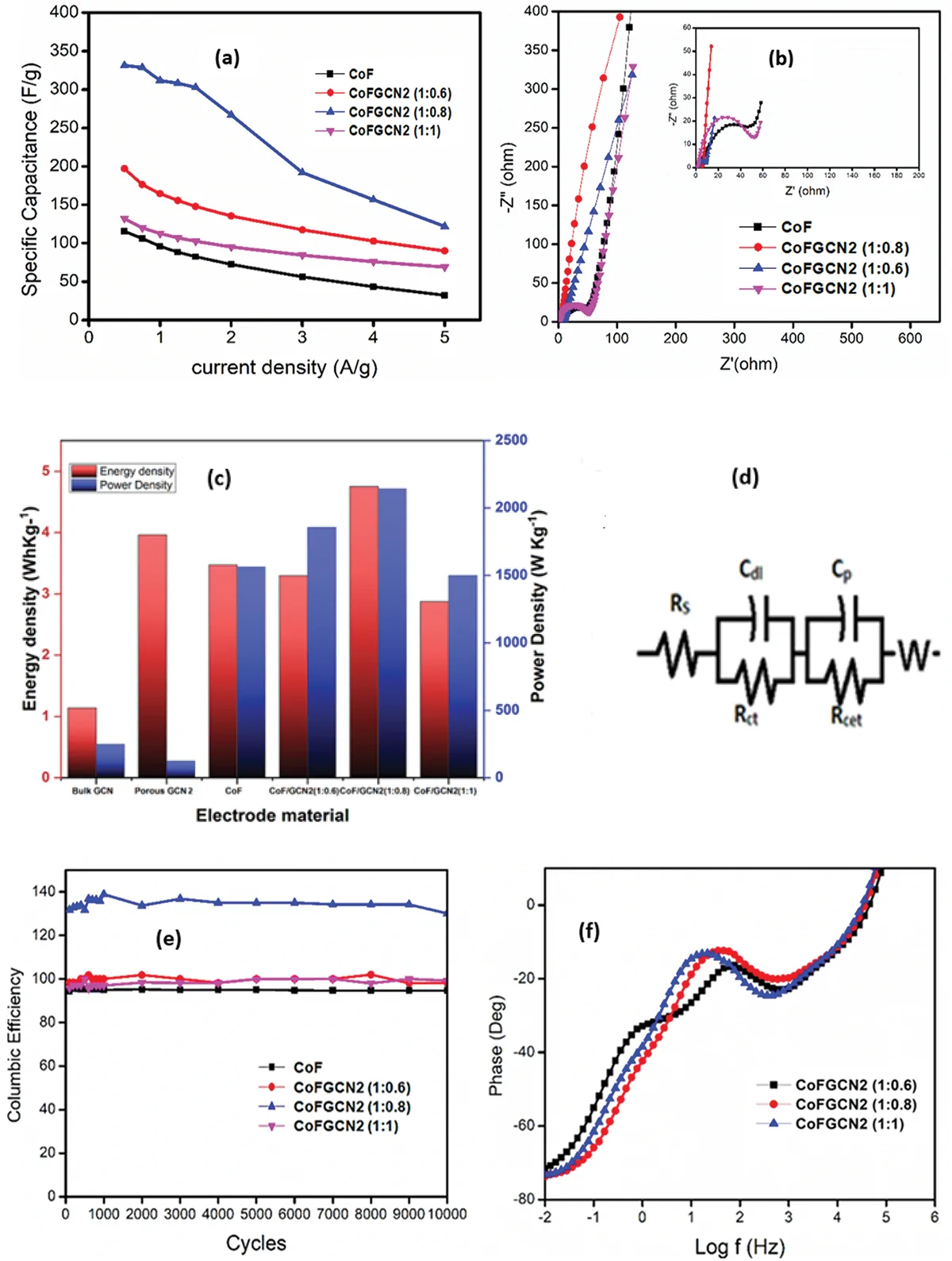
(a) Specific capacitance vs. Current density for GCN, CoF, and CoF/GCN2 electrodes from 0.5 Ag^−1^ to 4 Ag^−1^; (b) Nyquist plot of CoF and CoF/GCN electrodes (inset: high- frequency region of EIS spectra; c) Bar chart representing energy density vs. power density of GCN and CoF/GCN2 electrode materials; (d) equivalent circuit model for CoF/GCN2B electrode, (e) long-term cycling performance: constant current charge-discharge comparison of CoF and CoF/GCN electrodes; (f) bode plots of CoF/GCN2 nanocomposite electrodes with varying mass ratios.

The increase in discharge time observed in Figure 10(a) with decreasing current density is attributed to enhanced electrolyte ion penetration into the electrode. This greater penetration expands the active surface area, leading to increased specific capacitance. The opposite effect is seen at higher current densities, where limited diffusion time restricts ion access to the electrode’s interior, causing a decrease in capacitance [43]. The improved capacitance of the CoF/GCN2 composite can be ascribed to the following factors: (1) Compared to conventional carbon materials, the nitrogen in GCN enhances electron donation, conductivity, and surface wettability. (2) GCN act as a support, preventing CoF aggregation and facilitating charge transfer. (3) The CoF and GCN structure promotes electrochemical conductivity by improving charge transport at the interface. Despite a decrease in specific capacitance from 331.51 Fg^−1^ to 191.5 Fg^−1^ at 2 Ag^−1^, the CoF/GCN2B electrode exhibits good rate performance, as evidenced by its 95% capacitance retention.

Electrochemical impedance spectroscopy (EIS) can provide a better understanding of the relationship between capacitive properties and ionic resistances in materials. The EIS spectrum (Nyquist plot) of the CoF/GCN2 composites, measured over a frequency range of 10 mHz to 100,000 Hz, is shown in Figure 10(b). The inset shows expanded view of the high frequency measurement range. Electrical impedance measurements reveal a consistent trend across all materials. At high frequencies, the graphs show a semicircle, which is related to the resistance electrons encountered when moving flanked by the electrode and the electrolyte (charge-transfer resistance, R_ct_). A larger semicircle diameter indicates a higher charge-transfer resistance. Interestingly, the plots transition to a straight line at low frequency regions. This suggests that at low frequencies, the rate-limiting step in these materials is the diffusion of ions within the electrolyte (mass transfer process). The CoF/GCN composite exhibits a significantly lower equivalent series resistance (R_S_) compared to pure GCN and CoF, which is crucial for achieving high power density [44]. Additionally, the charge transfer resistance (R_ct_), determined from the high frequency semicircle in electrochemical impedance spectroscopy, is detailed in Table 3. The Warburg region (W), reflecting electrolyte ion diffusion in the low-frequency range, demonstrates that the CoF/GCN2B hybrid electrode exhibits a significantly improved ion diffusion process. The almost straight line of the CoF/GCN2B hybrid electrode shows a better ion diffusion process compared to the bulk GCN and CoF. The ZSimpWin 3.21 software was used to analyze the resistive and capacitive elements based on the well-fitted electrical equivalent circuit shown in Figure 10(d). The inset of Figure 10(b) displays a magnified impedance map in the high-frequency region. The electrical equivalent circuit consists of solution resistance (Rs), charge transfer resistance (Rct), electron transfer resistance (Rect), double-layer capacitance (Cdl), and pseudocapacitance (Cp). The EIS results for the CoF/GCN2B composite presented in Table 3 demonstrate reduced R_S_, R_ct_ and W values. These results corroborate the findings from the CV and GCD measurements and highlight its exceptional capacitance behavior. The enhanced electrochemical performance of CoF/GCN2B in comparison to other mass ratios is due to several factors. Firstly, the optimized mass ratio in CoF/GCN2B likely leads to an ideal balance between conductivity and electrochemical stability, enhancing charge transfer and ion diffusion. The increased conductivity, possibly due to the incorporation of CoF to GCN, facilitates better electron flow, while the specific structural properties of CoF/GCN2B may provide a higher surface area and more accessible active sites for ion adsorption. Additionally, the synergistic interaction between CoF and GCN may enhance charge storage capabilities, resulting in higher capacitance and improved cycling stability compared to other mass ratios. This combination of factors leads to superior performance in terms of energy and power density, making CoF/GCN2B a more efficient material for electrochemical applications such as supercapacitors (Table 4).

**Table 3.** Estimated values of solution resistance, charge transfer resistance, and Warburg impedance of CoF, and CoF/GCN composites electrode materials from EIS investigations.

CoF/GCN Composites (ratio)	Rs(Ω)	Rct(Ω)	Rect(Ω)	Cp	Cdl	W(Ω)
CoF	1.011	1.750	1684	0.017	0.09	0.069
CoFGCN2A (1:0.6)	1.355	2.890	1.777	0.1506	0.001	0.04
CoFGCN2B (1:0.8)	1.240	1.315	2.08	14.87	0.005	0.08
CoFGCN2C (1:1)	1.417	36.30	2.117	0.106	0.002	0.066

**Table 4.** Comparison of CoF/GCN2 and other CoF hybrid electrodes.

Electrode Materials	Electrolyte (M) KOH	Current density (Ag^−1^)	Method	Specific Capacitance (Fg^−1^)	Cycle life	Ref.
CoFe_2_O_4_	1	0.5	Solution combustion	125	–	[48]
CoFe_2_O_4_/rGO	2	0.5	Solvothermal	187	98% after 2000 cycles	[49]
CoFe_2_O_4_/rGO	6	0.5	One-pot solvothermal	356	87% after 5000 cycles	[48]
CoFe_2_O_4_/ CNF	2	0.5	Hydrothermal	188.36	–	[50]
CoFe_2_O_4_/ Carbon	5	0.16	Precipitation	102.5	81.6%, after 6000 cycles	[51]
CoFe_2_O_4_/g-C_3_N_4_	2	0.5	Sol-gel combustion	331	92% after 10,000 cycles	**This work**

The power density and energy density of the GCN and CoF/GCN2B nanocomposite electrode materials are depicted in the bar chart in Figure 10(c) [45]. It is evident that CoF/GCN2B attains an energy density of 4.75 Whkg^−1^ at a power density of 2142.8 Wkg^−1^.

The calculations for energy density and power density are based on following equations

(3) $$\mathrm{Energy}\;\mathrm{density}=\frac{0.5C_{s}X\Delta V}{3.6}\left(\mathrm{WhK}g^{-1}\right)$$

(4) $$\mathrm{Power}\;\mathrm{density}=\frac{\mathrm{Energy}\;\mathrm{density}\times 3600}{\Delta t}$$

Where C_s_(F/g) is the specific capacitance, ΔV (V) is the potential range, ϑ (mVs^−1^) is the scan rate and Δt (s) is the discharging time. The cycle performance of the electrode material is shown in Figure 10(e). After 10,000 cycles, the CoF/GCN2B shows no significant capacitance loss, maintaining a 95% capacitance retention rate at a current density of 6 Ag^−1^ and a 99.98% coulombic efficiency.

Understanding the Bode plot of a supercapacitor is critical for designing circuits that take advantage of its unique properties, particularly in energy storage systems [46]. This plot demonstrates how a supercapacitor will function at various frequencies, allowing for optimal integration into a wide range of electronic systems. The phase angles at 0 Hz for CoF/GCN2A, CoF/GCN2B, and CoF/GCN2C was −71, −77, −68 degrees, respectively as illustrated in Figure 10(f). This implies that CoF/GCN2B is closest to an ideal capacitor. Phase angles that are close to −90 degrees typically indicate high capacitive performance and a quick charge-discharge cycle.

Electric double-layer capacitance (EDLC) and Faradaic pseudo capacitance combine to store charge in the CoF/GCN2 composite. In alkaline conditions, CoF exhibits pseudocapacitive behavior due to reversible redox reactions between Co^2+^/Co^3+^ and Fe^2+^/Fe^3+^ couples. This makes it easier to transfer charge. The well-defined redox peaks in the CV curves confirm this pseudocapacitive behavior, consistent with similar cobalt-based hybrid electrode systems reported in the literature [9,47]. The GCN matrix improves EDLC through ion adsorption/desorption while increasing electrical conductivity with its nitrogen-rich structure. The synergistic interaction between CoF nanoparticles and GCN nanosheets plays a key role in enhancing the overall electrochemical performance, CoF provides rich redox-active sites while GCN improves electronic conductivity, surface wettability, and ion accessibility. This complementary coupling between Faradaic and non-Faradaic charge storage mechanisms is proposed as the primary reason for the superior capacitance and rate capability of CoF/GCN2B over its individual components. The observed CV, GCD, and EIS data demonstrate that the synergistic effect enhances the cycling stability, specific capacitance, and electrochemical kinetics of the composite electrode.

## 4. Conclusions

Cobalt ferrite (CoFe_2_O_4_) nanoparticles were prepared using a green synthesis approach, employing honey as a mediating agent in a sol-gel auto-combustion process. The CoFe_2_O_4_/g-C_3_N_4_ hybrid composite electrodes with weight ratios of 1:0.6, 1:0.8, and 1:1 was synthesized for supercapacitor applications. XRD data identifies the cubic spinel phase of CoFe_2_O_4_ and FTIR spectra suggest the presence of g-C_3_N_4_ within the composite material. The average crystallites size of hybrid nano structure was found to be 8 nm. The composite electrode displayed superior capacitance retention, stability, and electrochemical performance compared to bare CoFe_2_O_4_ nanoparticles. The CoFe_2_O_4_/g-C_3_N_4_ composites show significantly improved capacitance, attributed to the synergistic effect and high-quality interface between the components, with the 1:0.8 weight ratio demonstrating superior performance. At a scan rate of 2 mV/s, they exhibit specific capacitances of 470 Fg^−1^ and 331 Fg^−1^ at 0.5 Ag^−1^. Additionally, they possess an energy density of 4.75 Whkg^−1^ and a power density of 2142.5 Wkg^−1^, with 80% capacitance retention after 10,000 cycles. The g-C_3_N_4_ support, which effectively prevents the aggregation of CoFe_2_O_4_ nanoparticles, is the primary reason for the composite’s exceptional cycling stability. These properties demonstrate the promise of CoFe_2_O_4_/g-C_3_N_4_ composites as future candidates for storage energy materials. The enhanced electrochemical properties can be attributed to the unique characteristics of cobalt ferrite, and g-C_3_N_4_’s high surface area, and porous nanostructure facilitating rapid ion diffusion within the electrode material. The use of honey as a green mediator aligns with sustainable nanoparticle synthesis practices and contributes to the formation of well-defined nanostructures with good electrochemical behavior in the resulting materials.

## Supplementary Material

Supplemental Material
