# Supplementary material for: Surface area and composition-engineered CoFe2O4/g-C3N4 hybrid electrodes for high-performance supercapacitors
Source: Sci Technol Adv Mater. 2026 Jul 16;27(1):2700203. doi: 10.1080/14686996.2026.2700203 (PMC13508556; doi:10.1080/14686996.2026.2700203)
Supplement: Supplementary file 1 [file TSTA_A_2700203_SM8541.docx]

**Supporting Information**

Divakara S. G.^a*^, Shwetha K. P.^b^, Mahesh B.^c^, Vishnumurthy, K. A ^a^.

^a^ Department of Chemistry, RV College of Engineering, Mysore Road, Bengaluru-560 059, Karnataka, India

^b^ Department of Physics, RV College of Engineering, Mysore Road, Bengaluru-560 059,

Karnataka, India

^c^ Department of Chemistry, JSS Academy of Technical Education, Dr. Vishnuvardhan Road, Bengaluru-560 060, Karnataka, India

*Correspondence to: Divakara S. G. (E-mail: divakarsg@rvce.edu.in)

**Fig. S1.** FTIR spectra of bulk GCN, GCN1 and GCN2 samples.

**Fig. S2.** a) N_2_ adsorption/desorption isotherms of bulk GCN, GCN1and GCN2 materials. b) Pore distribution of bulk GCN, GCN1 and GCN2 materials.
